# Improving opioid substitution therapy in the acute hospital setting: implementation of a best practice guideline

**DOI:** 10.1136/bmjoq-2026-004153

**Published:** 2026-07-07

**Authors:** Michael Trent Herdman, Brihitejas Patel, Poppy Pierce, Vedika Apte, Zainab Mashal Hussain Wasti, James Norman, Agnes Niemet, Kate Robinson, Iona Martin, Marisha Wickremsinhe, Nerissa Tilouche, Adrian 'Bean’ Noctor, Jennifer Scott, Dan Lewer, Adam Holland, Magdalena Harris, Michael Brown

**Affiliations:** 1 University College London Hospitals NHS Foundation Trust, London, UK; 2 University College London Medical School, London, UK; 3 Central and Northwest London Hospitals NHS Foundation Trust, London, UK; 4 London School of Hygiene and Tropical Medicine, London, UK; 5 University of Bristol Centre for Academic Primary Care, Bristol, UK; 6 Bradford Teaching Hospitals NHS Foundation Trust, Bradford Institute for Health Research, Bradford, UK; 7 University of Bristol School of Psychological Science, Bristol, UK; 8 School of Population Health Sciences, University of Bristol Medical School, Bristol, UK

**Keywords:** Quality improvement, Hospital medicine, Patient-centred care

## Abstract

**Background:**

Delayed provision of opioid substitution therapy (OST) in acute hospitals contributes to patient distress, opioid withdrawal and discharge against medical advice (DAMA). We aimed to improve access to opioid substitution therapy (OST) for hospital inpatients, using the iHOST (‘improving hospital opioid substitution therapy’) clinical guidelines, developed through a deliberative consensus process in partnership with clinical, academic and peer experts with professional body oversight.

**Methods:**

This pre–post quality improvement study evaluates implementation of the iHOST guideline at a tertiary hospital serving inner city London, UK between 2021 and 2023. Clinical records of all patients administered OST in the 12 months before and 11 months after the policy’s introduction were evaluated for measures of process (reducing urine drug screening, improving time-to-administration), balancing (avoiding emergency naloxone administration, avoiding increased mortality) and outcome (discharge against medical advice).

**Results:**

Among 259 patients pre-policy and 194 post-policy, the median time to first methadone dose fell from 18.1 hours to 14.8 hours and the proportion of patients waiting more than 24 hours reduced from 32% to 21% (OR 0.57, 95% CI 0.37 to 0.88). Urine drug screening was one-sixth as likely to be performed on admission. There was no change in the likelihood of discharge against medical advice, which occurred for 24% of admissions during both periods (p=0.96). Naloxone was required for only 2% of admissions (five pre-policy, four times post p=1.00), indicating no compromise of patient safety.

**Conclusions:**

Implementing the iHOST guideline successfully reduced unnecessary delays to methadone administration without increasing the risk of opioid toxicity. While high rates of DAMA, even after improving access to OST, point to an ongoing need for patient-centred engagement strategies, successful implementation of the iHOST guideline supports its wider use: it is now under evaluation in two additional hospitals, after which it will be optimised to assess its utility for nation-wide practice.

**WHAT IS ALREADY KNOWN ON THIS TOPIC**

**WHAT THIS STUDY ADDS**

- This evaluation demonstrates successful implementation of the best-practice guideline developed by the improving hospital opioid substitution therapy (iHOST) project. This guideline removed reliance on urine drug screening and reduced delays in methadone administration without an increase in adverse events such as emergency naloxone use.

**HOW THIS STUDY MIGHT AFFECT RESEARCH, PRACTICE OR POLICY**

- Having demonstrated the feasibility and safety of implementation across a large hospital trust, future work will assess generalisability across additional iHOST research sites and further explore interventions to reduce discharge against medical advice, in co-development with patients and practitioners.

## Introduction

 People who use illicit opioids experience high rates of emergency hospital admission, often to address chronic health issues, injuries and infections.^1^,^5^ Fear of opioid withdrawal during hospital admission may lead people to delay seeking treatment^6 7^ and to leave hospital before treatment completion,^8^ resulting in emergency readmission, more expensive medical care and a greater risk of all-cause mortality.^9^,^12^ Hospital-based provision of opioid substitution therapy (OST)—which, in UK hospitals, commonly uses methadone—involves either continuing prescriptions in place in the community or initiating a new regimen for people with opioid dependence who do not receive community OST. Such provision can prevent opioid withdrawal, thereby fostering patient-centred care and enabling treatment completion.^13^

The National Institute for Health and Care Research-funded project ‘Improving Hospital Opioid Substitution Therapy’ (iHOST)^14^ aims to improve access to OST in hospital through the development, implementation and evaluation of a multicomponent intervention. A review of policies across acute hospital trusts in England has demonstrated significant variability and consequently, multiple procedural barriers to prompt OST provision.^15^ This has been observed in other countries including the USA and Australia, leading to efforts to standardise and improve policy.^16 17^ The iHOST project systematically developed a best practice guideline for opioid withdrawal management in England in collaboration with University College Hospitals, London (UCLH).^18^

This evaluation assesses the impact of the new guideline on key components of safe care for people with opioid use disorder at UCLH (time taken to provide methadone to patients, the need for any emergency opioid reversal and the incidence of discharge against medical advice, DAMA). It is part of a wider evaluation of the iHOST programme, which will also involve qualitative research and a quantitative analysis that includes additional iHOST and control hospital sites.

## Methods

### Guideline implementation

This pre–post quality improvement study was conducted at UCLH, a tertiary public hospital trust serving inner-city London. The iHOST best practice clinical guideline, implemented 15 November 2022, is included as online supplemental file 1. Key elements of the new iHOST guideline that differed from the previous UCLH guideline are published elsewhere,^18^ and include removal of mandatory urine drug screening prior to OST; increasing initial methadone titration dose; and recommending a higher day 1 titration dose when specific safety criteria are met. Discharge planning to ensure continuity of community care and reduce risk of opioid overdose is emphasised, with allowance for bridging prescriptions of OST and dispensing naloxone for future harm reduction at the time of hospital discharge. Guideline implementation was accompanied by intensive and repeated in-person training of doctors, nurses and pharmacists. This training included (1) introduction of ‘smartphrase’ and ‘order set’ shortcuts in the electronic health record to make OST prescribing and record-keeping more consistent and efficient; (2) systematic evaluation for signs of withdrawal using the Clinical Opioid Withdrawal Scale; (3) advice on subsequent dose titration; and (4) access to the e-learning iHOST training component on understanding opioid withdrawal and providing patient-centred care for people who use opioids.

### Data collection

The UCLH Trust Information Team identified the electronic records of all patient admissions during which methadone was prescribed (including date and time of admission, methadone prescription, first methadone dose administration and discharge) covering the 12 months before and 11 months after guideline implementation. Multiple admissions for the same patient were treated as separate episodes. Admissions where methadone was prescribed for reasons other than OST (such as complex or palliative pain management) were excluded. While the guideline addresses both methadone and buprenorphine prescribing, this evaluation focuses on methadone, as records were identified from electronic prescribing and whereas most methadone prescriptions are for OST, most buprenorphine prescriptions are for complex pain management, with infrequent use for OST.

The project team reviewed the Epic electronic health record (Epic Systems Incorporation, Madison, USA) for each admission, identifying key outcomes and indicators from the admission clerking, the medicines administration record and the discharge summary and supplemented this with a search of clinical notes for key phrases (‘Naloxone’, ‘methadone’, ‘opioid’) using the Epic System search functions to complete a clinical record form (online supplemental file 2). The team recorded each patient’s age, sex and admitting clinical team; whether the patient was receiving OST in the community at the time of admission; whether the patient was continuing to use unprescribed opioids, such as heroin, at the time of admission (in the judgement of the admitting clinical team); whether administering or delaying administration of OST was included in the admitting clinician’s plan; whether the automated smartphrase was used (in the post-iHOST period) to link the clinician to the prescribing guideline; whether the dose of methadone was adjusted after the initial prescription; the number of doses administered and omitted during the admission; whether the drug and alcohol liaison nurse specialist (DALN) team was consulted during the admission; whether naloxone was administered; whether urine toxicology was requested in the first 48 hours of admission; whether the patient discharged against medical advice; and whether the patient was known to have died at the time of review of the clinical record (and, if so, the date of death). Records of naloxone administration were validated by a second investigator.

For each encounter, one investigator performed the initial data extraction, consulting with senior colleagues as needed for clarification of ambiguous cases. After an initial search of 10 records each, the team reconvened to ensure consistent practices in data extraction. Where the classification of a patient was unclear after review of the records by a first investigator, a second, senior investigator reviewed the records to guide a consensus.

### Data description and analysis

Key process measures of steps toward successful OST were: (1) reduction in urine toxicology screening to confirm methadone use (as a practice that could delay OST administration); (2) median time from the decision to admit the patient to administration of the first dose. Key balancing measures were (3) avoidance of an increase in emergency administration of naloxone after commencing OST; (4) avoidance of an increase in 90-day mortality. The intended outcome measure was a reduction in hospital admissions ending in DAMA and associated re-admissions among patients receiving OST. These measures were captured and compared before and after guideline introduction.

For a wider description of changing practices surrounding OST, we also assessed clinical teams’ practices in planning to give or delay OST at the time of admission, use of the Epic OST smartphrase to guide methadone prescriptions, consultations with the DALN team, adjustments to the dose of OST during the admission and avoidance of unnecessary obstacles to OST delivery, such as requirements for consultation with community pharmacies and drug treatment services when out of office hours or inappropriate urine drug screening laboratory testing prior to commencing OST.

We compared the demographics and clinical characteristics and management of admissions in the pre-iHOST and post-iHOST periods. Patterns of opioid use at the time of admission (in terms of existing community OST prescriptions and ongoing unprescribed opioid use) were categorised. For assessments of delays in OST administration, admissions to critical care were excluded (on the basis that critical illness or sedation would likely introduce clinically appropriate delays to administration of OST).

We used the Standards for Quality Improvement Reporting Excellence (SQUIRE) reporting guideline in drafting and reporting this evaluation, included as online supplemental file 3.^19^

### Statistical analysis

Statistical analysis was undertaken using Stata MP V.13.0 (StataCorp, Texas, USA). Categorical variables were compared for admissions that did or did not end in DAMA and across the pre-iHOST and post-iHOST periods, by χ^2^ test (or Fisher’s exact test for comparisons involving fewer than five exposures or outcomes), and continuous variables were compared by Mann-Whitney U test. P charts were used to identify trends in the proportion of patients with delayed methadone administration.^20^

Multivariable logistic and Cox’s regression model was constructed to assess the association between the iHOST policy period and DAMA in the context of other variables associated with DAMA. Potential confounders assessed for the model were age category, gender, admitting clinical team, existing community OST prescription and ongoing use of unprescribed opioids at the time of admission. Effect modification was sought by bivariable analysis with the Mantel-Haenszel test and incorporated into the model if found.

### Patient and public involvement

The aim of the iHOST project—to improve opioid withdrawal management in National Health Service (NHS) hospitals—is directly informed by the priorities and experiences of people who use opioids. People with current and former experience of opioid use have been involved in the overarching iHOST project from conception, and two people with lived experience sit on the iHOST study advisory board. The iHOST peer expert group (six members) also meet every 3 months to oversee and discuss study progress. Core intervention components were co-produced in workshops with people who use opioids. The study principal investigator (MTH) and patient and public involvement lead (AN) both have extensive experience of opioid use and OST. MTH’s prior research with this population found that poor OST provision in hospitals is a primary barrier to accessing and receiving care.^6 15^ The iHOST intervention was developed in collaboration with people who use opioids through qualitative interviews and workshops at the UCLH site, and this evaluation’s outcome measure—time from admission to administration of OST—is informed by patient priorities, given the relevance this has to onset of withdrawal and associated patient distress.^14^ The patient and public involvement lead (AN) played a key role in recruitment at the site for iHOST qualitative interviews and workshops with NHS healthcare providers. Findings of this evaluation have been discussed with the peer expert group, who are actively involved in study dissemination activities including resource development.

## Results

### Population characteristics

**Figure 1:**
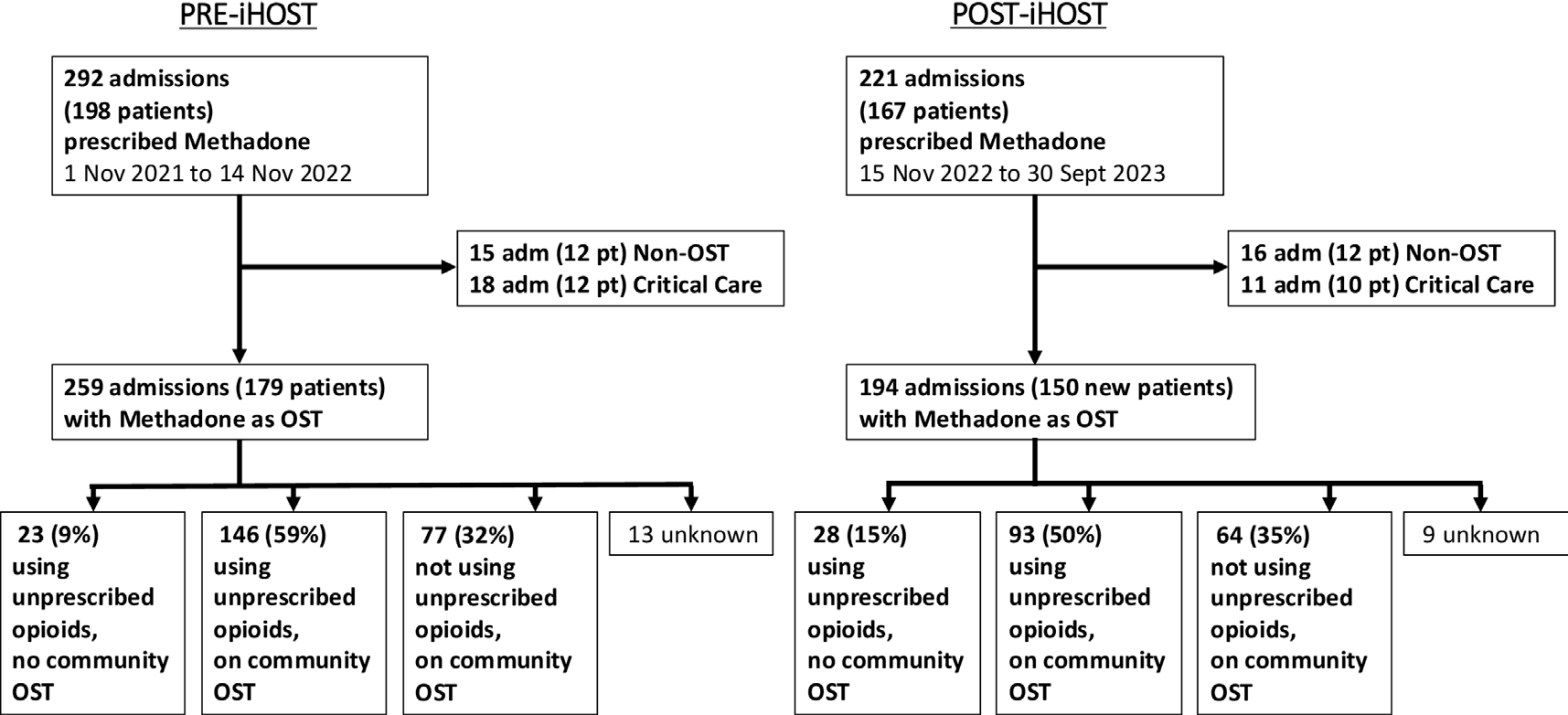
Admissions identified and included in the analysis. adm, admissions; iHOST, improving hospital opioid substitution therapy; OST, opioid substitution therapy; pt, patients.

In the study period leading up to implementation of the iHOST policy from 1 November 2021 to 14 November 2022, methadone was prescribed for 292 admissions to UCLH for 198 individual patients. 15 admissions (12 patients) had methadone prescribed for reasons other than opioid withdrawal management and were excluded from further analysis. A further 18 admissions (12 patients) were to critical care and were excluded from the main analysis (as sedation status, which might appropriately postpone OST, could not be readily ascertained). 259 admissions (179 patients admitted between 1 and 13 times) were prescribed methadone for OST on medical and surgical wards and were included (mean of 4.8 admissions per week). In the post-iHOST period from 15 November 2022 to 30 September 2023, methadone was prescribed for 221 admissions (167 patients), with 16 admissions (12 patients) excluded as non-OST and 11 admissions (10 patients) excluded as critical care. 194 admissions (150 patients) were prescribed methadone for OST on medical and surgical wards and were included (mean of 4.3 admissions per week; figure 1).

The demographics of admitted patients were similar through the pre-iHOST and post-iHOST periods (table 1). 36% were female and 64% male; median age was 51 years, with an IQR of 41–59 and range of 23–78. The largest proportion of patients were admitted under the acute/general medical service (46%); the next largest specialist medical team was infectious diseases (18%). 55% of patients had a community OST prescription and were also using unprescribed opioid drugs, such as heroin. 33% had a community OST prescription and were no longer using unprescribed opioids; 12% were using unprescribed opioids and had no community OST prescription.

**Table 1:** Baseline characteristics of admissions of patients receiving opioid substitution therapy (n=453)

Characteristic	Pre iHOST n	(%)	Post iHOST n	(%)	Total n	(%)
	259		194		453	
Female	97	(37)	66	(34)	163	(36)
Age category: <30 years	8	(3)	5	(3)	13	(3)
30–39	51	(20)	38	(20)	89	(20)
40–49	60	(23)	42	(22)	102	(22)
50–59	83	(32)	54	(28)	137	(30)
60–69	48	(19)	47	(24)	95	(21)
70+	9	(3)	8	(4)	17	(4)
Treating team*						
Acute general medicine	125	(48)	85	(44)	210	(46)
Infectious diseases	42	(16)	38	(20)	80	(18)
Other medical	44	(17)	34	(18)	78	(17)
Surgical	48	(19)	37	(19)	85	(19)
Opioid use category†						
Using unprescribed opioids, no community OST	23	(9)	28	(15)	51	(12)
Using unprescribed opioids, community OST	146	(59)	93	(50)	239	(55)
Not using unprescribed opioids, community OST	77	(31)	64	(35)	141	(33)
Unknown/missing	13	.	9	.	*22*	.
Deceased at time of data collection	55	(21)	25	(13)	80	(18)

* Excludes admissions directly to critical care.

† Opioid use category at the time of admission, ascertained from the notes of the admitting clinical team.

iHOST, improving hospital opioid substitution therapy; OST, opioid substitution therapy.

### Practices in prescribing OST and managing opioid dependence

**Figure 2:**
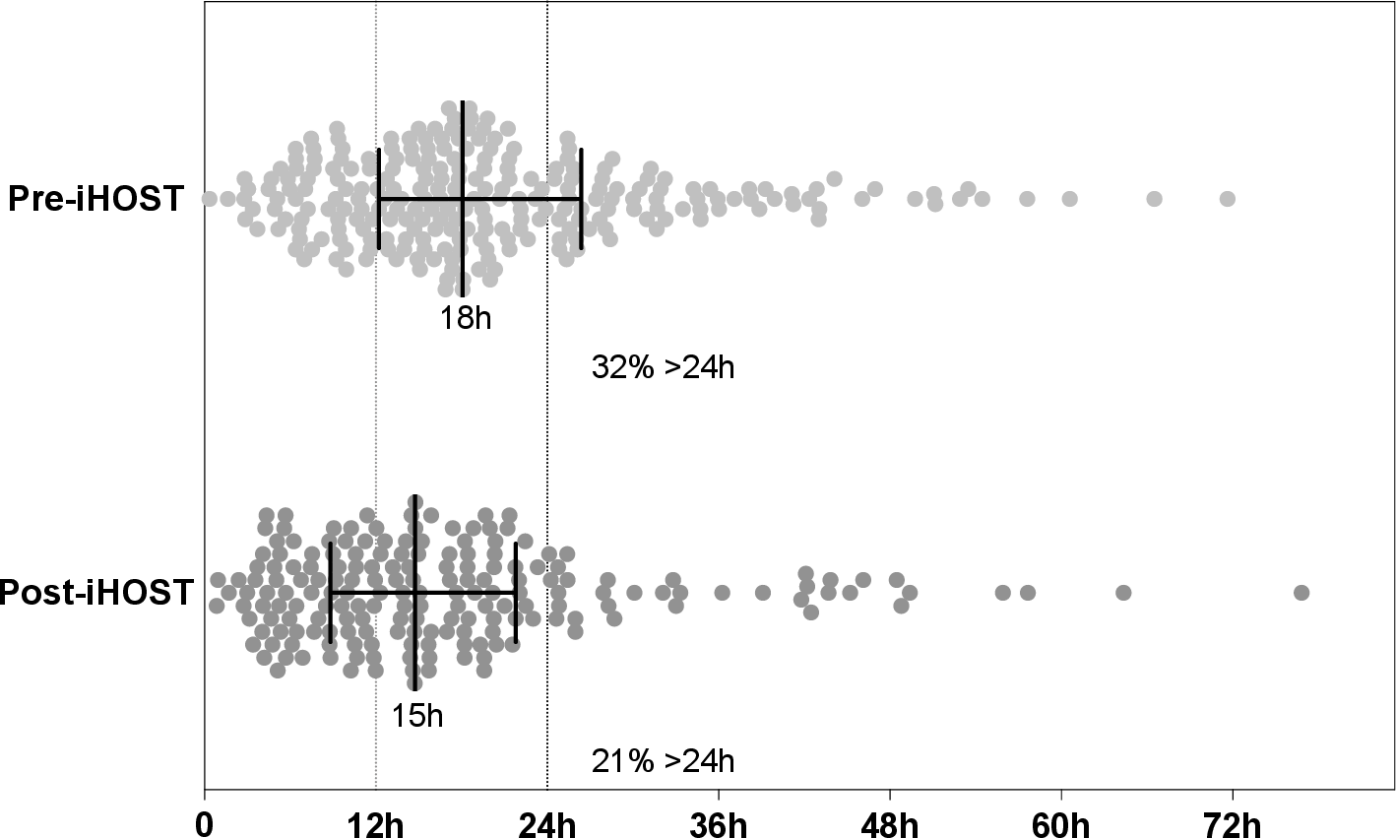
Time from admission decision to OST administration in the pre-iHOST and post-iHOST periods. Whisker plot shows the median and IQR for each group. iHOST, improving hospital opioid substitution therapy; OST, opioid substitution therapy.

With implementation of the iHOST policy, the median time from the decision to admit a patient to the administration of the first dose of methadone fell from 18.1 hours (IQR 12.2–26.4 hours) to 14.8 hours (IQR 8.8–21.8 hours; p<0.001). The proportion of patients waiting for longer than 24 hours for methadone was reduced from 32% to 21% (OR 0.57, 95% CI 0.37 to 0.88; figure 2).

**Figure 3:**
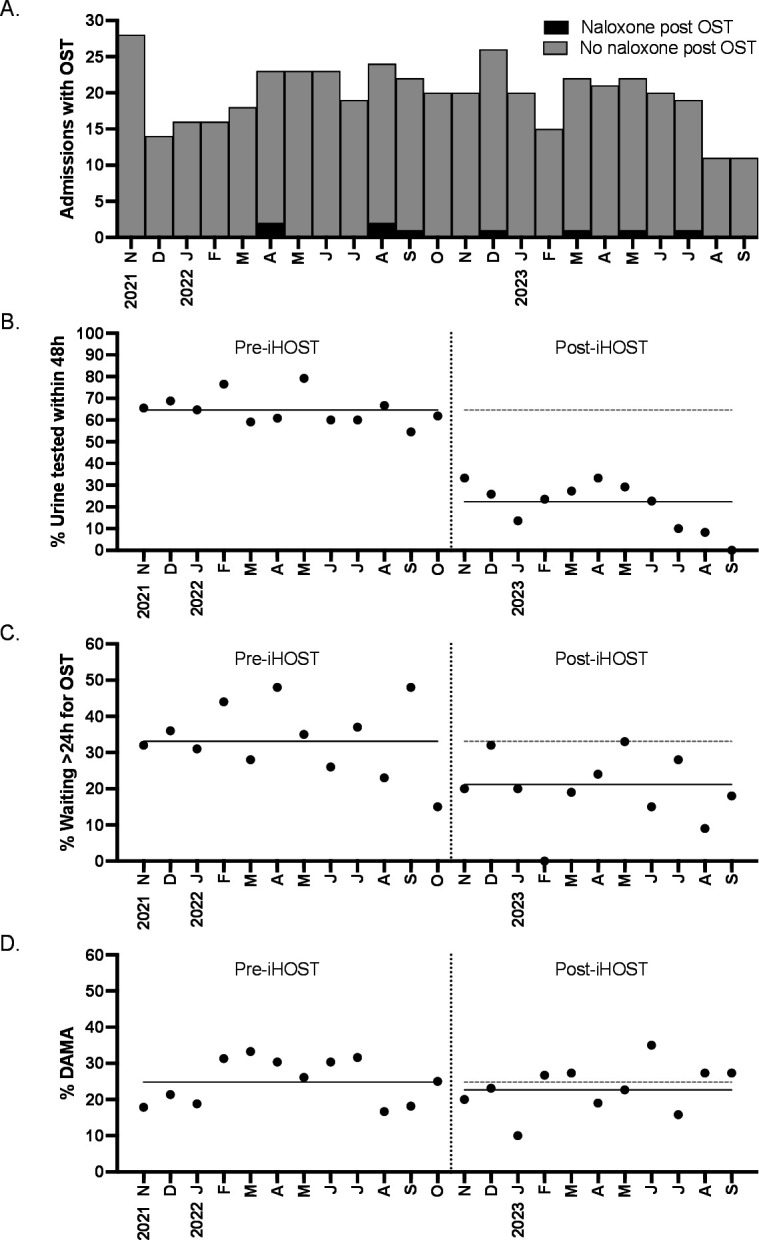
Process indicators and outcomes in the pre-intervention and post-intervention periods. Panel A: Monthly admissions with OST administration and naloxone administration in the pre-iHOST and post-iHOST periods. Grey bars: admissions with OST administration and no subsequent naloxone administration. Black bars: admissions with OST administration followed by naloxone administration. Dotted line: official implementation of iHOST policy (from 15 November 2022). Panel B: Monthly urine drug screening on admission. Points: % patients with urine specimens collected for urine drug screening within 48 hours of the decision to admit. Solid line: mean % tested for the period; dashed line: pre-iHOST period’s mean for reference in the post-iHOST period. Panel C: Monthly delays to OST administration. Points: % patients waiting more than 24 hours from the decision to admit to administration of the first OST dose. Solid line: mean % for the period. Dashed line: pre-iHOST period’s mean for reference in the post-iHOST period. Panel D: Monthly DAMA. Points: % admissions ending in DAMA among patients receiving OST. Solid line: mean % DAMA for the period. Dashed line: pre-iHOST period’s % DAMA for reference in the post-iHOST period. DAMA, discharge against medical advice; iHOST, improving hospital opioid substitution therapy; OST, opioid substitution therapy.

Urine drug screening tests reduced substantially after iHOST implementation (67% pre-iHOST falling to 25% post-iHOST; OR 0.17, 95% CI 0.11 to 0.26). In the post-iHOST period, fewer urine drug screens were in the first 48 hours of admission (the time frame during which they were likely to be the basis for OST prescribing; 96% of tests pre-iHOST and 77% post-iHOST; figure 3 Panel B).

Among patients prescribed methadone, OST was explicitly mentioned in the admitting team’s recorded management plan at the time of admission in 87% of cases in the pre-iHOST period and 78% in the post-iHOST period (OR 0.51, 95% CI 0.31 to 0.85), potentially indicating its incorporation into the routine practice of prescribing regular medications. Explicit decisions to delay OST (for any reason) were less common post-iHOST period, falling from 27% to 16% (OR 0.50, 95% CI 0.31 to 0.81). Over the course of admissions, patients had their methadone doses adjusted from the initial prescription in a comparable proportion of cases: 37% pre-iHOST and 44% post-iHOST (OR 1.35, 95% CI 0.92 to 1.98). In the post-iHOST period, the smartphrase introduced to electronic guidance on the dosing of methadone at the time of prescribing was used for 15% of admissions. It was used more often for patients with ongoing use of unprescribed opioids (22% vs 6%, OR 4.8, 95% CI 1.6 to 14.7). The DALN Team was consulted during more admissions post-iHOST (55% pre, 66% post; OR 1.61, 95% CI 1.09 to 2.37).

10 of 453 admissions (4 pre-iHOST and 6 post-iHOST) ended with the patient’s death (2% pre, 3% post, p=0.268), and 25 patients (15 pre, 10 post) died within 90 days of discharge (6% pre, 5% post, p=0.769). 80 patients (18%) had died by the time of data collection approximately 6 months after the end of the study period: 55 from the pre-iHOST period (21%) and 25 (13% from the post-iHOST period (while drawing a direct comparison would be inappropriate as more time had elapsed since the pre-iHOST period, this underlines the high mortality risk faced by this patient cohort). Only a small number of patients were provided with a discharge prescription for naloxone for harm reduction and prevention of future overdoses (two patients in the pre-iHOST period and six post-iHOST).

### Use of naloxone

Naloxone was administered in the emergency management of patients after commencing OST on nine occasions during the study: five before implementation of the iHOST policy (2% of admissions), with no evidence of increased likelihood of its use post-implementation (four administrations, 2% of admissions, figure 3 Panel A). Five patients (three pre-iHOST, two post-iHOST) who were treated with naloxone post OST provision had become drowsy after being absent from the ward or away from staff, during which time clinical teams suspected they had taken unprescribed opioids. The median time to post-OST naloxone administration was 2 days from admission (ranging from the day of admission to day 6). No patients who received post-OST emergency naloxone died during their hospital stay in either the pre-intervention or post-intervention period.

### Timelines of delayed OST and discharge against medical advice

Attribute control charts were used to describe the temporal relationship between the overall patterns of clinical performance and the introduction of the iHOST guideline. Following iHOST implementation, a sustained shift in process measure performance was observed: the proportion of patients undergoing urine toxicology testing fell below the pre-intervention median in all months observed (figure 3 Panel B), and the proportion of patients waiting over 24 hours for OST administration stayed below the pre-intervention median for 10 of 11 months (figure 3 Panel C), satisfying standard run chart rules for a non-random improvement.^20^ After iHOST implementation, the outcome measure—proportion of admissions ending in DAMA—changed little, from 25% to 23%, with no evident shift in monthly observations (figure 3 Panel D).

DAMA occurred a median of 3 days after admission (IQR 2–7 days, range 0–37 days). 43% of DAMAs occurred within 3 days of the decision to admit, and 74% occurred within 7 days. Admissions ending in DAMA occurred with equivalent frequency in the pre-iHOST and post-iHOST periods; they were more common among patients with ongoing unprescribed opioid use (with three times the odds of DAMA, OR 3.12, 95% CI 1.75 to 5.55), and among patients under 40 (with five times the odds of DAMA of patients 60 and older, OR 5.02, 95% CI 2.54 to 9.93; table 2). We found no evidence of a change in the likelihood of admissions ending in DAMA (adjusted OR 1.07, 95% CI 0.67 to 1.73) or in the hazard of DAMA over the course of admissions (adjusted HR 0.99, 95% CI 0.67 to 1.47) using logistic and Cox’s regression models to compare post-iHOST and pre-iHOST periods, adjusting for age, gender, admitting specialty, community OST and ongoing opioid use.

**Table 2:** Characteristics associated with discharge against medical advice among admissions with opioid substitution therapy administered

Characteristic	Category	Discharge against medical advice n	Total admissions n	(%)	Crude OR	(95% CI)	P value
iHOST period	Pre	62	259	(24)	.	.	.
	Post	46	194	(24)	0.99	(0.64 to 1.53)	0.955
Age category	60+ years	18	112	(16)	.	.	.
	40–59	40	239	(17)	1.05	(0.88 to 1.93)	.
	Under 40	50	102	(49)	5.02	(2.54 to 9.93)	<0.001*
Gender	Male	63	290	(22)	.	.	.
	Female	45	163	(28)	1.37	(0.88 to 2.14)	0.159
Principal specialty	Acute medicine	48	210	(23)	.	.	.
	Infection	29	80	(36)	1.92	(1.09 to 3.37)	0.021
	Surgery	17	85	(20)	0.84	(0.45 to 1.57)	0.592
	Other medical	14	78	(18)	0.74	(0.38 to 1.43)	0.369
Community OST	No	18	53	(34)	.	.	.
	Yes	88	393	(22)	0.56	(0.30 to 1.04)	0.064
Ongoing unprescribed	No	17	143	(12)	.	.	.
Opioid use	Yes	87	294	(30)	3.12	(1.75 to 5.55)	<0.001

* χ2 test for trend.

iHOST, improving hospital opioid substitution therapy; OST, opioid substitution therapy.

## Discussion

We successfully introduced a new opioid substitution therapy best practice guideline to an acute London Hospital Trust, improving access to timely medication and providing a patient-centred approach to care. Implementing the guideline led to a substantial reduction in time from the decision to admit a patient to receipt of methadone, with the proportion of patients waiting for longer than 24 hours nearly halved. While the median time to first methadone dose was still nearly 15 hours, this is in the context of a patient cohort in which most patients were receiving community OST and many would have taken their daily methadone dose prior to hospital attendance. For patients who have received community OST shortly before attendance, administration up to 24 hours after admission might be appropriate. The guideline’s successful implementation, as is evident from reduced requests for urine drug screening (which the study team has previously identified as an important obstacle to OST^18^) and increased referrals to the DALN team, reflects a high level of clinical fidelity and acceptability among staff. This was achieved without evident adverse consequences, as demonstrated by the unchanged—and low—rate of naloxone administration.

The guideline implemented as part of the iHOST study in November 2022 remains UCLH policy. Our evaluation findings to date indicate no need to revert to former practice. Further work to sustain improved OST provision will need to focus on the challenges common to all acute hospital settings, such as incorporating best practice into the orientation and training of new and rotating staff, engaging with specialist teams for whom OST is a less frequent patient need and building responsiveness to withdrawal symptoms into the nursing workflow. In addition, elements of the guideline remain incompletely realised–including prescription of naloxone at discharge for future harm reduction in the community, which has been the focus of ongoing interventions in the trust since the period evaluated here.

This pragmatic evaluation has some key limitations. The absence of a contemporaneous control group means we cannot definitively exclude the possibility that the trends we observed were due to external factors (including access to opioids in the community or contemporaneous changes to community outreach services), and not to the guideline itself. However, process measures examining the implementation context suggest it functioned as planned in improving access to OST.

Additionally, our analysis was restricted to patients who received OST potentially overlooking the high-risk population who left hospital before a prescription could be initiated. If the new guideline succeeded in delivering OST to this population earlier, we may have underestimated its impact by restricting the analysis to patients who received at least one dose of methadone.

Multiple admissions for the same patient were analysed as independent episodes—meaning that frequently admitted patients contribute proportionally more to the analysis. These patients may bear the greatest cumulative risk of DAMA and subsequent adverse outcomes, and restricting the analysis to their first admissions would disproportionately exclude them. As this is an evaluation of the quality of care, we included all admissions rather than restricting to one per patient, since each admission represents a discrete opportunity to provide timely and appropriate OST.

The stability of patient demographics and opioid use categories (table 1) and the consistent admission volumes (4.8 vs 4.3 per week) in the pre-intervention and post-intervention periods suggest that the cohorts remained clinically comparable over time, providing reassurance that the inability to capture patients who did not receive OST and the decision to include multiple episodes for repeatedly admitted patients were not substantial sources of differential bias.

We did not find evidence that the iHOST guideline reduced discharges against medical advice in its first 11 months of implementation. Interventions, particularly in complex hospital systems, require time to embed. Although we found a positive impact on time from admission to provision of OST, improved withdrawal management might take time to impact DAMA rates. This would only be evident with a commensurate evaluation of patient data over future years of implementation. In our sample, DAMA was most common in those using unprescribed opioids and in over half of cases, it occurred more than 3 days after OST initiation (the period during which most symptoms of withdrawal would be expected). This suggests drivers other than acute withdrawal management contributed to DAMA at this stage of hospital admission. Qualitative process evaluation data can provide a nuanced understanding of the contexts in which DAMA occurs and inform opportunities for further intervention to reduce the risk to patients dependent on opioids.

The ongoing iHOST research programme includes implementation of the intervention in two other UK acute hospital settings to assess generalisability and extend quantitative and qualitative analysis to a wider context. Outcomes of this ongoing work will inform recommendations for a national best practice guideline.

## Supplementary material

10.1136/bmjoq-2026-004153 online supplemental file 1

10.1136/bmjoq-2026-004153 online supplemental file 2

10.1136/bmjoq-2026-004153 online supplemental file 3

## Data Availability

Data are available upon reasonable request.
