# Supplementary material for: Improving opioid substitution therapy in the acute hospital setting: implementation of a best practice guideline
Source: BMJ Open Qual. 2026 Jul 7;15(3):e004153. doi: 10.1136/bmjoq-2026-004153 (PMC13343018; doi:10.1136/bmjoq-2026-004153)
Supplement: online supplemental file 1 [file bmjoq-15-3-s001.pdf]

Prevention and treatment of opioid withdrawal in hospital

See Full Guideline for further information
See Flowsheet (appendix 1) and Clinical Opiate Withdrawal Scale (COWS) (appendix 2)

DIAGNOSIS AND CAUSES

Use ***.methadoneOST*** or ***.buprenorphineOST***

1. Establish opioid dependency
   - Community opioid substitution therapy (OST) Rx
   - Regular heroin use (frequency, amount, route)
   - History of opioid withdrawal symptoms
2. Conduct physical examination
   - Opioid withdrawal symptoms using clinically validated scoring tool, e.g., **COWS** (**Appendix 2**)
   - Polysubstance use (NB: alcohol withdrawal is a medical emergency; see local guidance [LINK])
3. For patients on community Rx:
   - Confirm medication, formulation, current dose, and whether consumption is supervised (community pharmacist/prescriber); confirm date of last consumption (pharmacist if supervised/patient if unsupervised)

NB: Re-titrate OST if last consumption reported as 3+ days from date of hospital admission

REQUEST

Monitor all patients for opioid toxicity four hours after each dose and then as per NEWS

- If RR<12, oxygen saturation below target, or reduced level of consciousness: withhold OST
- **If unresponsive: administer naloxone**

ADVICE, REVIEWS & REFERRALS

- Inform Drug and Alcohol Liaison CNS of all patients prescribed OST in hospital: 07415331353
- Liaise with Community Drug Treatment (CDTS) service for all patients prescribed OST in hospital
- See section in full guideline on acute pain management

DISCHARGE

- Administer full dose OST on day of discharge
- Prescribe **TTA naloxone (Prenoxad)**
- **Patients admitted on community Rx**: Arrange continuation of OST Rx with CDTS (*OOH bridging supply not permitted if not pre-agreed with CDTS*)
- For patients initiated on OST in hospital: Arrange urgent appointment with CDTS for day of discharge

MEDL GUIDELINE DETAILS
Authors: iHOST team (M Harris, A Holland, J Scott & M Wickremsinhe), M Brown, J Norman **MEDL Editor:** M George.
Specialist: Drug and Alcohol Team **Pharmacist:** Agnes Niemet

CGC approval: 11/10/22 **Review date: 11/10/25**

TREAT

- **Rx naloxone PRN for all patients on OST**
- NB: Do not prescribe OST if contraindications: head injury, acute respiratory depression, coma (see BNF)
- You **DO NOT** have to send a urinary Tox Screen before prescribing OST

Continuing community Rx

Use ***.methadoneOST*** or ***.buprenorphineOST***

- Rx usual dose once daily (BD dosing if cautions, patient preference, or to enable pain management)
- ***For patients on methadone***: monitor for symptoms of withdrawal; if withdrawal symptoms persist, prescribe 5-10mg methadone PRN; max. daily dose increase 10mg, max. weekly dose increase 30mg

Initiating/re-titrating methadone (1mg/1ml)

(use ***.methadoneOST*** smartphrase)

DAY ONE

- Rx 20mg starting dose methadone
- Monitor for symptoms of withdrawal 4-hourly
- Rx additional 10mg PRN methadone 4-hourly up to 40mg total day-one dose
- If withdrawal symptoms persist, prescribe up to 60mg total day-one dose **under expert supervision**

DAY TWO ONWARDS

- Convert total day-one dose into daily prescription and Rx in divided doses (BD)
- Monitor for withdrawal symptoms; if withdrawal symptoms persist:
  - Increase dose by up to 10mg PRN every other day (max. weekly dose increase of 30mg over day-one dose); If day-one dose ≤40mg, can increase dose by up to 10mg on day two

Initiating/re-titrating buprenorphine

(use ***.buprenorphineOST*** smartphrase)

DAY ONE

- **NB: Only administer buprenorphine when withdrawal symptoms are present**
- Rx 4mg buprenorphine
- Monitor for withdrawal symptoms 4-hourly; if withdrawal symptoms persist, Rx additional 2mg PRN (up to 8mg total day-one dose)

DAY TWO ONWARDS

- Convert total day-one dose into daily prescription and Rx once daily
- Monitor for withdrawal symptoms; Rx additional 4mg dose 4-hourly if required, up to 8mg total PRN (max. 16mg total day-two dose)

Medl

Appendix 1

Prevention and treatment of opioid withdrawal in hospital

1. Patient assessment for opioid dependence

- Establish use: patient history of drug use (community OST prescription and/or heroin use)
- Establish tolerance: medication, formulation, dose, frequency of pick-up, supervised/unsupervised (OST); amount, frequency, route, and duration of use (other opiates)
- Physical examination for withdrawal symptoms, e.g. using the Clinical Opioid Withdrawal Scale (Appendix 3)

NB: Do not prescribe OST if contraindications: head injury, acute respiratory depression, coma (see BNF).

Evidence of opioid dependence?

- No → Do not prescribe OST; monitor for symptoms of withdrawal
- Yes → On a community OST prescription?
  - No → 3. Initiating OST in hospital
  - Yes → Dose confirmed (prescriber/pharmacist); last consumption within 3 days (patient if unsupervised Rx / pharmacist if supervised Rx)
    - No → 3. Initiating OST in hospital
    - Yes → 2. Continuing community OST prescription

2. Continuing community OST prescription

- Rx naloxone PRN
- Inform CDTS of admission; discuss changes in dosing and discharge planning

Day one

- Rx usual dose once daily (BD if cautions or if preferred by patient)
- Monitor withdrawal symptoms 4-hourly
- [Methadone only]: if withdrawal symptoms persist, Rx additional 5-10mg PRN methadone (max. 10mg/day) to cover on-top opioid use

Day two onwards

- [Methadone only] Adjust usual dose to include any additional methadone and convert into daily dose; max. weekly dose increase 30mg

For patients whose dose cannot be promptly confirmed: See Box 3; once dose confirmed, Rx any additional OST needed to meet total usual dose; Rx usual daily dose from following day

3. Initiating OST in hospital

- Inform local CDTS to facilitate referral on discharge

Methadone (1mg/1ml)

Day one

- Rx naloxone PRN
- Rx initial dose 20mg (split dosing if cautions); consider 30mg with skilled clinical judgement
- Rx additional dose 10mg PRN 4-hourly up to 40mg total day-one dose if withdrawal symptoms persist
- If withdrawal symptoms persist 4 hours after 40mg dose, consider additional 10mg dose 4-hourly up to max. 60mg total day-one dose under expert supervision (p. 13)

Day two onwards

- Convert total day-one dose into daily prescription and Rx in divided doses (BD)
- Monitor for withdrawal symptoms; Rx additional 10mg/day every other day (maximum 30mg increase over seven days); If day-one dose ≤40mg, can increase dose by 10mg on day two

Buprenorphine

Day one

- Rx naloxone PRN
- Rx 4mg buprenorphine (NB: only administer initial dose buprenorphine when withdrawal symptoms are present)
- Monitor for withdrawal symptoms 4-hourly; if withdrawal symptoms persist, Rx additional 2mg PRN (up to 8mg total day-one dose)

Day two onwards

- Convert total day-one dose into daily prescription and Rx once daily
- Monitor for withdrawal symptoms; Rx additional 4mg dose 4-hourly if required, up to 8mg total PRN (max 16mg total day-two dose)

Monitor all patients for signs of opioid toxicity 4 hours after each dose and then as per NEWS

- If RR<12, oxygen saturation below target, or if reduced level of consciousness, call ward doctor urgently

4. Acute pain management (see page 15)

5. Discharge

Patients admitted on community Rx	Patients initiated on OST in hospital
• Rx OST on day of discharge; Rx TTA naloxone<br>• Contact CDTS to continue/re-start community Rx<br>• Where agreed with CDTS, Rx TTA OST if discharged out-of-hours to prevent break in OST	• Rx OST on day of discharge; Rx TTA naloxone<br>• Arrange urgent appt. with CDTS<br>• Arrange bridging prescription to prevent break in OST if same-day appt. unavailable

Clinical Opiate Withdrawal Scale (COWS)

Resting pulse rate (beats/minute)
0 - Pulse rate 80 or below
1 - Pulse rate 81-100
2 - Pulse rate 101 -120
4 - Pulse rate greater than 120

Sweating (over past ½ hour, not accounted for by room temperature or client activity)
0 - No chills of flushing
1 - Patient report of chills or flushing
2- Flushing or observable moistness on face
3 - Beads of sweat on brow or face
4 - Sweat streaming off face

Restlessness (observation during assessment)
0 - Able to sit still
1 - Reports difficulty sitting still, but is able to do so
3 - Frequent shifting or extraneous movements of legs/arms
5 - Unable to sit still for more than a few seconds

Pupil dilation
0 - Pupils pinned or normal size for room light
1 - Pupils possibly larger than normal for room light
2 - Pupils moderately dilated
5 - Pupils so dilated that only the rim of the iris is visible

Bone or joint aches (not attributable to other health conditions or presenting complaint)
0 - Not present
1 - Mild diffuse discomfort
2 - Client reports severe diffuse aching of joints/muscles
4 - Client is rubbing joints or muscles and is unable to sit still because of discomfort

Rhinorrhoea or lacrimation (not accounted for by cold symptoms or allergies)
0 - Not present
1 - Nasal stuffiness or unusually moist eyes
2 - Nose running or tearing
4 - Nose constantly running or tears streaming down cheeks

Vomiting, nausea, and/or diarrhoea (over last ½ hour)
0 - No GI symptoms
1 - Stomach cramps
2 - Nausea or loose stool
3 - Vomiting or diarrhoea
5 - Multiple episodes of diarrhoea or vomiting

Tremor (observation of outstretched hands)
0 - No tremor
1 - Tremors can be felt, but not observed
2 - Slight tremors observable
4 - Gross tremor or muscle twitching

Yawning
0 - No yawning
1 - Yawning once or twice during assessment
2 - Yawning three or more times during assessment
4 - Yawning several times per minute

Gooseflesh skin
0 - Skin is smooth
3 - Piloerection of skin can be felt or hairs standing up on arms
5 - Prominent piloerection

Scoring: "Mild"— 5 to 12; "Moderate"—13 to 24; "Moderately severe"—25 to 36; "Severe"—more than 36

This MEDL guideline is intended to support decision making by trained doctors and must be interpreted appropriately in the clinical context. It covers management for **adult patients only**. The authors cannot be held responsible for their use. Only current on date printed, refer to myUCLH for definitive version.
