# Supplementary material for: Improving opioid substitution therapy in the acute hospital setting: implementation of a best practice guideline
Source: BMJ Open Qual. 2026 Jul 7;15(3):e004153. doi: 10.1136/bmjoq-2026-004153 (PMC13343018; doi:10.1136/bmjoq-2026-004153)
Supplement: online supplemental file 2 [file bmjoq-15-3-s002.pdf]

Quality Improvement Project: Prevention and treatment of opioid withdrawal in hospital. (v.1.2)

Protocol for Data Collection and Analysis

M Trent Herdman, Brihitejas Patel, Michael Brown

Background

People who use opioid drugs have a high rate of morbidity and mortality. Opioid use poses risks of infection, injury, and predisposition to chronic health conditions, and people who use opioids face disproportionate risks in accessing and completing treatment for these conditions.

Fear of opioid withdrawal is a common obstacle to seeking care, and experiencing withdrawal in hospital frequently leads to self-discharge against medical advice before treatment is completed. Premature discharge increases the likelihood of readmission—potentially requiring more complex care—and is associated with increased all-cause mortality.

Opioid withdrawal in hospital can be prevented by providing timely, adequate opioid substitution therapy (OST) in the form of oral methadone or sublingual buprenorphine. Providing correctly dosed OST promotes an alliance between patients and health carers, supporting patients to remain in hospital for the duration of care, and enabling clinicians to provide other aspects of treatment.

Over the course of 2023, UCLH implemented new clinical guidelines to promote the prevention and treatment of opioid withdrawal by supporting medical, nursing, and pharmacy staff in providing OST, encompassing steps to promote:

- Patient assessment to identify the need for OST in patients with community prescriptions, ongoing illicit opioid use, or both;
- Systematic evaluation for signs of withdrawal using the clinical opioid withdrawal scale (COWS) and advice on titrating the dose accordingly;
- Avoidance of unnecessary barriers to OST prescribing, such as mandated confirmation of community prescriptions or urine toxicology results;
- Communication with patients and community pharmacies to establish pre-hospital regimens and plan recommencement at discharge;
- Prescribing naloxone to ensure availability in the event of opioid toxicity during the admission, and provision of take-home naloxone on discharge to reduce the risk of overdose in the community.

Interventions to promote these goals included:

- Publication of the full OST policy and MEDL guideline for rapid reference;
- Training for staff at educational meetings and through e-learning platforms;
- Development of smartphrases in EPIC to guide prescribers through key aspects of OST provision;
- Incorporation of recommendations into EPIC order sets for prescribed medication;
- Communication to stakeholders through UCLH newsletters.

Objectives:

In order to improve the prevention and treatment of opioid withdrawal for patients hospitalized at UCLH, this quality improvement project seeks to evaluate clinical objectives and process indicators over the first year of the OST guideline's implementation, to develop interventions to improve provision of OST, and to develop plans for prospective monitoring these objectives in future.

Primary Endpoint:

- **Proportion of patients prescribed OST who discharge against medical advice;**

Key Process Indicators:

For each patient prescribed OST, we will assess:

- **Time from decision to admit patient to prescription of OST;**
- **Time from prescription of OST to administration of first dose;**
- **Dose omissions of prescribed OST during the admission;**
- **Administration of Naloxone (as an indicator of potential OST-related adverse events);**

Additionally, we will seek to identify whether interventions intended to promote adequate and timely OST dosing were used to guide the patient's care:

- Use of an EPIC OST smartphrase to generate a note guiding prescription of methadone or buprenorphine;
- Consultation of the Drug and Alcohol Liaison Nurse team;
- Use of EPIC smart order sets in prescribing OST;
- Adjustment of OST dosing during the admission;
- Avoiding inappropriate mandate for confirmation of community prescription before commencing OST;
- Avoiding inappropriate mandate for urine toxicology before commencing OST.

We will seek to target future educational activities by identifying which clinical teams patients requiring OST were admitted under, and which teams exhibited particularly high or low rates of achieving key indicators;

We will look for evidence of downstream effects of the OST policy, by assessing provision of take-home naloxone at the time of discharge, rates of re-admission, and rates of mortality following admission.

Retrospective Assessment of Data from 2023:

The Trust's Data team will extract a linelist of all patients receiving OST, along with the dates and times of admission, OST prescription, first OST dose administration, and discharge. These data will be used to calculate delays in prescription and administration.

Where a patient experienced multiple admissions, data will be collected for each admission.

The QIP Team will use the list of all admissions to guide access to clinical records, identifying key endpoints and indicators from the relevant EPIC encounter. It is anticipated that this will require reading the admission clerking, the medicines administration record (MAR), and the discharge summary, and supplementing this with a search of clinical notes for key phrases (“Naloxone”, “DALN”) using the EPIC search box. After an initial search of 5-10 records each, the team will reconvene to share tips for finding key endpoints, and to ensure consistent practices in data extraction.

Endpoints will be summarised on a monthly timeline to identify any trends in achieving processes, and to guide analysis of where obstacles are still encountered, and how the quality of OST provision can be improved in future.

Outcome measures for OST Quality Improvement Project: Clinical Record Form (v.1.1)

Underlined text reflects data to be entered into the shared Excel line list.
Italicized text is guidance on how to find this information and complete the record.

Assessors may wish to use this document to collect data while reviewing the EPIC record, or enter data directly into the Excel line list.

Go to the Encounter that corresponds with the Admission Date, then find the Admission Clerking (the most complete note from the admitting team which describes the reason for admission and plan for management), the MAR Report (list of all doses of drugs prescribed and administered, and the Discharge Letter/TTO (the summary of the admission at the time of discharge, transfer, or death). You will also need to use the search box on the left of the screen to find notes using the OST Smartphrase, notes about DALN (Drug and Alcohol Liaison Nurse) consultations, and administration of Naloxone.

For each patient on the list of identified recipients of OST, we hope to capture the following information:

QIP Record Number:
MRN:
Date of Admission:
Age: *In years at the time of admission*
Sex: *As recorded in EPIC*

1. **Was the patient prescribed Methadone/Buprenorphine as Opioid Substitution Therapy?**
   Yes/No/Unclear

(If prescribed for a reason other than OST—such as complex pain relief/palliative care—say so in the notes column and stop here: there is no need to complete the remainder of the evaluation.)

2. **Under which team was the patient admitted?**
   Name of team or teams
   eg: acute medicine; orthopedics; joint care acute medicine and orthopaedics.

3. **a. Was patient identified in the Admission Clerking as potentially needing OST?**
   Yes/No/Unclear
   If unclear, describe why in the final "Notes" column of the Excel line list.
   Any mention of a plan to prescribe OST, methadone, or buprenorphine, or to seek more information about community OST provision, should be scored as "yes."
   b. Was there a planned delay in starting OST noted in the Admission Clerking?

<u>Yes/No/Unclear</u>

For example, because the patient was drowsy, intoxicated, or had recently taken OST in the community prior to admission.

4. **Was the OST smartphrase used to generate a note including advice on dosing?**
   <u>Yes/No/Unclear</u>
   If the smartphrase has been used, it will likely appear as a separate note in EPIC, including detailed guidance on dosing and communication with pharmacies. The text can be found by searching for "Prescribing methadone as opiate substitution therapy" in the search box (this phrase is in the note's heading).

5. **Was the dose of OST subsequently adjusted?**
   <u>Yes/No/Unclear</u>
   This should be apparent by reviewing the MAR Report for the dates of the admission.

6. **a. How many doses were administered?**
   <u>Number of doses</u>
   This should be apparent by reviewing the MAR report (doses appear in green and show "given" if you hover over them)
   b. How many doses were omitted/not administered?
   This can be found by counting the prescribed doses not in green (hover over them to see reasons, and add up all prescribed doses not administered).

7. **Was the Drug and Alcohol Liaison Nurse consulted for advice or review?**
   <u>Yes/No/Unclear</u>
   Consider using search box for "DALN" to identify notes.

8. **Was naloxone administered during the admission?**
   <u>Yes/No/Unclear</u>
   Check the MAR (which shows when the last dose of any administered drug was given), and confirm using the search box for "Naloxone"

9. **Was urine toxicology requested?**
   <u>Yes/No/Unclear</u>
   Check under the Results tab over the dates of the admission, and confirm using the search box for "toxicology"

10. **Was the patient discharged with Naloxone to reduce risk of overdose?**
    <u>Yes/No/Unclear</u>
    Check the discharge summary.

11. **Did the patient discharge against medical advice? [*Principal Outcome Measure*]**
    <u>Yes/No/Unclear</u>
    Check the discharge summary. Confirm by using the search box for "self-discharge" and "discharge against medical advice."

12. **Is the patient now known to be deceased?**

Yes/No

13. **If yes, date of death**
    DD/MM/YYYY
    Epic will display the date of death for any deceased patients in the patient summary information on the left of the screen.
14. **Notes on Data Entry**
    Include any notes on fields that were unclear, or other issues encountered in finding or evaluating information.
15. **Notes on Clinical Case**
    Include a sentence or two on the reason for admission, and any observations about obstacles to care or reasons for discharge against medical advice.

Outcome measures automatically captured by the data team and pre-entered into the spreadsheet:
Date/time of decision to admit
Date/time of first OST prescription
Date/time of first OST administration
Date/time of discharge
