# Supplementary material for: Improving opioid substitution therapy in the acute hospital setting: implementation of a best practice guideline
Source: BMJ Open Qual. 2026 Jul 7;15(3):e004153. doi: 10.1136/bmjoq-2026-004153 (PMC13343018; doi:10.1136/bmjoq-2026-004153)
Supplement: online supplemental file 3 [file bmjoq-15-3-s003.docx]

The SQUIRE reporting checklist

For checking that reports that describe systematic work to improve the quality, safety and value of healthcare, using a range of methods to establish the association between observed outcomes and interventions. can be understood and used by everyone

|  | Item Description | Location (or reason for not reporting) |
| --- | --- | --- |
| **Title and Abstract** |  |  |
| [1. Title](https:/resources.equator-network.org/reporting-guidelines/squire/items/title.html?utm_source=squire&utm_medium=checklist&utm_campaign=SQUIRE_2_0_2016_v1_1) | Indicate that the manuscript concerns an initiative to improve healthcare (broadly defined to include the quality, safety, effectiveness, patient-centredness, timeliness, cost, efficiency and equity of healthcare). | Title (p.1) |
| [2. Abstract](https:/resources.equator-network.org/reporting-guidelines/squire/items/abstract.html?utm_source=squire&utm_medium=checklist&utm_campaign=SQUIRE_2_0_2016_v1_1) | 2a. Provide adequate information to aid in searching and indexing.  2b. Summarise all key information from various sections of the text using the abstract format of the intended publication or a structured summary such as: background, local problem, methods, interventions, results, conclusions. | 2a: Abstract and keywords (p.1).  2b: Abstract (p.1). |
| **Introduction** |  |  |
| [3 & 4. Problem description & Available Knowledge](https:/resources.equator-network.org/reporting-guidelines/squire/items/problem-description-available-knowledge.html?utm_source=squire&utm_medium=checklist&utm_campaign=SQUIRE_2_0_2016_v1_1) | 1. Nature and significance of the local problem. 2. Summary of what is currently known about the problem, including relevant previous studies. | 3. Introduction: Paragraph 1 (p.2).  4. Introduction: Paragraph 2 (p.2). |
| [5. Rationale](https:/resources.equator-network.org/reporting-guidelines/squire/items/rationale.html?utm_source=squire&utm_medium=checklist&utm_campaign=SQUIRE_2_0_2016_v1_1) | Informal or formal frameworks, models, concepts, and/or theories used to explain the problem, any reasons or assumptions that were used to develop the intervention(s), and reasons why the intervention(s) was expected to work. | Introduction Paragraph 2 (p.2). |
| [6. Specific aims](https:/resources.equator-network.org/reporting-guidelines/squire/items/specific-aims.html?utm_source=squire&utm_medium=checklist&utm_campaign=SQUIRE_2_0_2016_v1_1) | Purpose of the project and of this report. | Introduction Paragraph 3 (p.2). |
| **Methods** |  |  |
| [7. Context](https:/resources.equator-network.org/reporting-guidelines/squire/items/context.html?utm_source=squire&utm_medium=checklist&utm_campaign=SQUIRE_2_0_2016_v1_1) | Contextual elements considered important at the outset of introducing the intervention(s). | Methods Paragraph 1 (p.3). |
| [8. Intervention(s)](https:/resources.equator-network.org/reporting-guidelines/squire/items/interventions.html?utm_source=squire&utm_medium=checklist&utm_campaign=SQUIRE_2_0_2016_v1_1) | 1. Description of the intervention(s) in sufficient detail that others could reproduce it 2. Specifics of the team involved in the work. | A. Methods Paragraph 1 and reference 15 (p.3).  B. Methods Paragraphs 2-4 (p.3-4). |
| [9. Study of the Intervention(s)](https:/resources.equator-network.org/reporting-guidelines/squire/items/study-of-the-interventions.html?utm_source=squire&utm_medium=checklist&utm_campaign=SQUIRE_2_0_2016_v1_1) | 1. Approach chosen for assessing the impact of the intervention(s) 2. Approach used to establish whether the observed outcomes were due to the intervention(s). | A. Methods Paragraphs 5-7 (p.4).  B. Methods Paragraphs 9-10 (p.4-5). |
| [10. Measures](https:/resources.equator-network.org/reporting-guidelines/squire/items/measures.html?utm_source=squire&utm_medium=checklist&utm_campaign=SQUIRE_2_0_2016_v1_1) | 1. Measures chosen for studying processes and outcomes of the intervention(s), including rationale for choosing them, their operational definitions, and their validity and reliability 2. Description of the approach to the ongoing assessment of contextual elements that contributed to the success, failure, efficiency, and cost. | A. Methods Paragraphs 5-7 (p.4).  B. Methods Paragraph 7 and references 13 and 15 (p.4). |
| [11. Analysis](https:/resources.equator-network.org/reporting-guidelines/squire/items/analysis.html?utm_source=squire&utm_medium=checklist&utm_campaign=SQUIRE_2_0_2016_v1_1) | 1. Qualitative and quantitative methods used to draw inferences from the data 2. Methods for understanding variation within the data, including the effects of time as a variable. | A. Methods Paragraphs 9-10 (p.4-5).  B. Methods Paragraph 9-10 (p.4-5). |
| [12. Ethical considerations](https:/resources.equator-network.org/reporting-guidelines/squire/items/ethical-considerations.html?utm_source=squire&utm_medium=checklist&utm_campaign=SQUIRE_2_0_2016_v1_1) | Ethical aspects of implementing and studying the intervention(s) and how they were addressed, including, but not limited to, formal ethics review and potential conflict(s) of interest. | Methods Paragraph 12 (p.5). |
| **Results** |  |  |
| [13 a & b. Evolution of the intervention and details of process measures](https:/resources.equator-network.org/reporting-guidelines/squire/items/intervention-evolution-process-measures.html?utm_source=squire&utm_medium=checklist&utm_campaign=SQUIRE_2_0_2016_v1_1) | 13a. Initial steps of the intervention(s) and their evolution over time (e.g., time-line diagram, flow chart, or table), including modifications made to the intervention during the project.  13b. Details of the process measures and outcome. | A. Figure 1 (p.13).  B. Results Paragraphs 3-5, Figures 2-3 (p.7-8, p.13). |
| [13 c, d & e Contextual elements and unexpected consequences](https:/resources.equator-network.org/reporting-guidelines/squire/items/context-unexpected-consequences.html?utm_source=squire&utm_medium=checklist&utm_campaign=SQUIRE_2_0_2016_v1_1) | 13c. Contextual elements that interacted with the interventions  13d. Observed associations between outcomes, interventions and relevant contextual factors  13e. Unintended consequences such as benefits, harms, unexpected results, problems or failures associated with the intervention(s) | C. Results Paragraphs 1-2, Table 1 (p.6-7).  D. Results Paragraphs 1-2 and Table 2. (p.6, p.10).  E. Results Paragraphs 6-7 (p.8). |
| [13 e. Missing data](https:/resources.equator-network.org/reporting-guidelines/squire/items/missing-data.html?utm_source=squire&utm_medium=checklist&utm_campaign=SQUIRE_2_0_2016_v1_1) | 13e. Details about missing data. | Figure 1, Table 1. |
| **Discussion** |  |  |
| [14. Summary](https:/resources.equator-network.org/reporting-guidelines/squire/items/summary.html?utm_source=squire&utm_medium=checklist&utm_campaign=SQUIRE_2_0_2016_v1_1) | 14a. Key findings, including relevance to the rationale and specific aims  14b. Particular strengths of the project | A. Discussion Paragraph 1 (p.11).  B. Discussion Paragraphs 1-2 (p.11). |
| [15. Interpretation](https:/resources.equator-network.org/reporting-guidelines/squire/items/interpretation.html?utm_source=squire&utm_medium=checklist&utm_campaign=SQUIRE_2_0_2016_v1_1) | 15a. Nature of the association between the intervention(s) and the outcomes  15b. Comparison of results with findings from other publications  15c. Impact of the project on people and systems  15d. Reasons for any differences between observed and anticipated outcomes, including the influence of context  15e. Costs and strategic trade-offs, including opportunity costs | A. Discussion Paragraphs 1, 4 (p.11).  B. Discussion Paragraph 4 (p.11).  C. Discussion Paragraph 4 (p.11).  D. Discussion Paragraph 4 (p.11).  E. Discussion Paragraphs 1-2 (p.11). |
| [16. Limitations](https:/resources.equator-network.org/reporting-guidelines/squire/items/limitations.html?utm_source=squire&utm_medium=checklist&utm_campaign=SQUIRE_2_0_2016_v1_1) | 16a. Limits to the generalisability of the work.  16b. Factors that might have limited internal validity such as confounding, bias, or imprecision in the design, methods, measurement, or analysis.  16c. Efforts made to minimise and adjust for limitations. | A. Discussion Paragraph 3 (p.11).  B. Discussion Paragraphs 3-4 (p.11).  C. Discussion Paragraph 3 (p.11). |
| [17. Conclusion](https:/resources.equator-network.org/reporting-guidelines/squire/items/conclusion.html?utm_source=squire&utm_medium=checklist&utm_campaign=SQUIRE_2_0_2016_v1_1) | 17a. Usefulness of the work  17b. Sustainability  17c. Potential for spread to other contexts  17d. Implications for practice and for further study in the field  17e. Suggested next steps | A. Discussion Paragraphs 1, 4 (p.11).  B. Discussion Paragraphs 2, 4 (p.11).  C. Discussion Paragraph 4 (p.11).  D. Discussion Paragraph 4 (p.11).  E. Discussion Paragraphs 2, 4 (p.11). |
| **Other information** |  |  |
| [18. Funding](https:/resources.equator-network.org/reporting-guidelines/squire/items/funding.html?utm_source=squire&utm_medium=checklist&utm_campaign=SQUIRE_2_0_2016_v1_1) | Sources of funding that supported this work. Role, if any, of the funding organisation in the design, implementation, interpretation and reporting. | Funding Statement (p.14). |
